# Exploring the Mechanism of Weikang Keli in Inhibiting Gastric Cancer through the MAPK Signaling Pathway: Based on Network Pharmacology and Experimental Verification

**DOI:** 10.1155/2022/2662288

**Published:** 2022-05-02

**Authors:** Xiang Shao, Yuping Liu, Cuihua Jiang, Yue Sun, Qiyang Zhang, Jialin Gu, Jiege Huo, Canhong Hu

**Affiliations:** ^1^Affiliated Hospital of Integrated Traditional Chinese and Western Medicine, Nanjing University of Chinese Medicine, Nanjing, Jiangsu 210028, China; ^2^Nanjing University of Chinese Medicine, Nanjing, Jiangsu 210046, China; ^3^Jiangsu Province Academy of Traditional Chinese Medicine, Nanjing, Jiangsu 210028, China

## Abstract

**Background:**

With a high incidence and limited treatments, gastric cancer (GC) seriously threatens human health worldwide. Weikang Keli (WK) is a compound prescription summed up from clinical experience. In our previous studies, WK has been proved to exert antitumor effects. However, there are no research studies to discuss and verify its mechanism as a compound.

**Objective:**

The aim of the study is to explore the potential molecular mechanism of WK in the treatment of GC with the aid of network pharmacology and verify it through experiments.

**Methods:**

Related databases were used to obtain genes and targets of WK and gastric cancer. A protein-protein interaction (PPI) network is constructed and visualized by Cytoscape 3.7.2. Gene ontology (GO) and Kyoto Encyclopedia of Genes and Genomes (KEGG) pathway enrichment analyses were used to analyze core targets. The cell viability of MFC and BGC-823 cells was determined by CCK8. Immunofluorescence was used to determine autophagy of GC cells. Moreover, the effect of WK on the MAPK signaling pathway in GC cells and tumor tissues of ICR mice was detected by Western blot.

**Results:**

A total of 106 cross targets of WK and GC were obtained. According to the enrichment analysis of GO and KEGG, we target the MAPK signaling pathway to discuss the mechanism of WK on GC. Cell experiments proved that WK inhibited the viability of gastric cancer cells in a dose-dependent and time-dependent manner. Autophagosome aggregation and an increase in the expression of an autophagy marker protein LC3-II can also be observed in WK groups. Further animal experiments showed that the tumor inhibition rate of WK showed a dose-effect relationship. Moreover, the expressions of p-JNK, p-p38, and p-ERR1/2 proteins in the MAPK signaling pathway in WK Group were downregulated both in the cell and animal experiments, compared with the blank control group.

**Conclusion:**

WK showed an explicit antitumor effect on gastric cancer through the MAPK signaling pathway, and the curative effect varies in different concentrations. Besides, in model mice, the antitumor effect of high-dose WK group is close to that of platinum. This study provided a theoretical basis for the application of WK in the clinical treatment of gastric cancer.

## 1. Introduction

With its incidence ranking fifth, gastric cancer is one of the most common cancers in the world. It is widely recognized that the top treatment pick for early gastric cancer is surgery. For advanced gastric cancer, chemotherapy containing platinum and fluorouracil is still the first-line treatment. Other therapies, such as HER2-targeted drugs (trastuzumab), anti-angiogenesis drugs (VEGF monoclonal antibody ramucirumab and immunotherapy), and PD1 monoclonal antibodies (nivolumab and pembrolizumab) also play a more and more important role in advanced gastric cancer. Nevertheless, the overall survival rates of treatments mentioned above are still limited, and it is difficult to achieve a balance between efficacy and adverse reactions, especially for patients in poor physical conditions [1].

Weikang Keli (WK), containing *Panax ginseng C.A.* Mey., *Atractylodes macrocephala* Koidz., *Arum ternatum* Thunb., *Curcumae rhizoma*, *Actinidia chinensis Planch,* and *Rhodiola rosea* L., is a compound prescription summarized from clinical experience. It works to invigorate spleen, invigorate blood, and disperse masses. In our previous research, we found that WK exerted antitumor effects by inducing autophagy to cause the death of gastric cancer SGC-7901 cells. [2] Although many studies proved that main ingredients of WK, such as *Panax ginseng C.A.* Meyer, [3] curcumin, [4–6] *Salidroside*, [7, 8] *Pinellia ternata* [9], participated in the antitumor process via PI3K/AKT, EGFR/STAT3, and wnt/*ß*-catenin, there was no further research to discuss its mechanism as a formulae.

As an emerging discipline, network pharmacology defined drugs work by regulating multiple proteins and participating in multiple signal pathways. Different from the previous “one-target, one-drug” mode, network pharmacology established a “network-target, multiple-component-therapeutics” mode.

Through the integration of genes, proteins, and pathways, a “drug-target-disease” network is established. Moreover, a series of algorithms are used to predict associations between protein complexes and infer molecular mechanisms. Supported by network pharmacology, a balance between efficacy and toxicity in the new drug research was obtained, which coincided with the overall concept of traditional Chinese medicine (TCM), and had a positive significance for verifying the mechanism of TCM, especially Chinese herbal compound prescriptions [10, 11].

This study explored the potential mechanism of WK against gastric cancer with the aid of network pharmacology and selected the potential pathway to conduct experimental verification, which provides more adequate evidence for expanding the application of this compound prescription.

## 2. Materials and Methods

### 2.1. Network Pharmacology-Based Screening of the Active Ingredients and Potential Targets of WK

We searched the compounds of WK in Traditional Chinese Medicine Systems Pharmacology Database and Analysis Platform (TCMSP, http://tcmspw.com/tcmsp.php), Chemistry Database [DB/OL] (http://www.organchem.csdb.cn.) and related papers to make a complement. PubChem (https://pubchem.ncbi.nlm.nih.gov/) was used to identify 2D structures of related compounds. The 2D structures were put into SwissADME (http://www.swissadme.ch/) for ADME (absorption, distribution, metabolism, and excretion) screening. ‘GI absorption' in pharmacokinetics should be ‘High' and more than 2 items should be ‘Yes' in drug-likeness (Lipinski, Ghose, Veber, Egan, Muegge). After ADME screening, active ingredients were finally selected. Then, the 2D structures of the selected compounds were imported into Swiss Target Prediction (http://www.swisstargetprediction.ch/) for target prediction. Duplicates in the target information were removed and the left ones were imported into Cytoscape 3.7.2 to construct the “active ingredient-target” network of WK.

### 2.2. Active Ingredient-Target for Gastric Cancer

With the keyword “gastric cancer”, gastric cancer-related targets in GeneCards (The Human Gene Database, https://www.genecards.org/) and DisGeNET (https://www.disgenet.org/) were searched. All targets are standardized according to Uniprot (https://www.uniprot.org/).

### 2.3. Construction of Protein-Protein Interaction (PPI)

Cross targets of WK and gastric cancer were obtained and were imported into String (https://www.string-db.org/) to achieve the relationships between proteins and visualize them via Cytoscape 3.7.2.

### 2.4. Enrichment Analysis of the Pathway

Metascape (https://metascape.org/) was conducted to conduct Gene Ontology (GO) and Kyoto Encyclopedia of Genes and Genomes (KEGG) pathway enrichment analyses. GO analysis includes biological process (BP), cellular component (CC), and molecular function (MF) analysis.

### 2.5. Molecular Docking

PDB was downloaded from RCSB Protein Data Bank (PDB, https://www.rcsb.org/) and mol2 formats of the active ingredients of the compound were downloaded from TCMSP. Autodock and Autodock Vina were used to calculate the docking scores between target proteins and molecules after they were dehydrated and hydrogenated. The interaction between proteins and bioactive compound should be stronger if its docking score is higher.

### 2.6. Reagents

Anti-p38 antibody (ab31828), anti-ERK1 + ERK2 antibody (ab54230), anti-PERK antibody (ab65142), *β*-actin Antibody (E12-051-1), anti-JNK1 + JNK2 + JNK3 antibody (ab179461), and other reagents were purchased from ABCam (United Kingdom), and fetal bovine serum was provided by GIBCO (United States).

WK consisting of *Panax ginseng C. A.* Mey. (10 g), *Atractylodes macrocephala* Koidz. (10 g), *Arum ternatum* Thunb. (10 g), *Curcumae rhizoma* (10 g), *Actinidia chinensis Planch* (15 g), and *Rhodiola rosea* L. (10 g) was provided by the traditional Chinese medicine (TCM) pharmacy of Jiangsu Province Hospital on Integration of Chinese and Western Medicine. The Chinese medicine was authenticated to be in compliance with the 2005 edition of “Chinese Pharmacopoeia”. WK crude drugs were crushed and soaked in the tenfold double distilled water for 24 h. Then, the mixture was heated, filtered, and refluxed for 2 cycles to get concentrated solution. The concentrate was evaporated at 60°C for the WK extract and the extract was stored at −20°C.

### 2.7. Cell Culture

The mouse gastric cancer cell line MFC and human gastric cancer cell line BGC-823 are provided by Nanjing Enjing Biotechnology Co., Ltd. MFC and BGC-823 cells were cultured in RPMI-1640 with 10% fetal bovine serum and 1% penicillin-streptomycin, under a humidified atmosphere at 37°C with 5% CO_2_.

### 2.8. Animals

ICR mice are provided by Nanjing Enjing Biotechnology Co., Ltd. For the experiment, mice were maintained in the SPF laboratory animal room of Jiangsu Province Hospital on Integration of Chinese and Western Medicine. The experiment was approved by Ethics Committee of Jiangsu Province Hospital on Integration of Chinese and Western Medicine (the ethical review registration number is AEWC-20170120-13) and complied with the regulations of Experimental Animal Ethics Committee.

### 2.9. Cell Viability Assay

EnoGeneCell™ Counting Kit-8 (CCK-8) was used to test cell viability. Cells were inoculated into a 96-well plate at a concentration of 1 × 10^5^ cells/mL and placed in a 37°C, 5% CO_2_ incubator for 24 hours. Then, the gastric cancer cells were treated by 100 *μ*L WK of different concentrations (0.39～200 mg/mL), and distilled water of equal dose was added as the control group. Each group had 5 replicate wells and was placed in a 37°C, 5% CO_2_ incubator for 72 hours. Then, the 10 *μ*L CCK-8 reagent was added to each well, incubation was continued for 4 hours in the incubator, the OD value was measured at 490 nm with a microplate reader, and GraphPad Prism v8.0.2.263 was used to calculate the cell inhibitory rate and IC_50_ value.

### 2.10. Immunofluorescence to Determine Autophagy of Cells

Stable eGFP-LC3 MFC cells were selected to establish a blank control group, WK groups (10 mg/mL, 20 mg/mL, 30 mg/mL, 40 mg/mL), and positive control group (50 nM Rapamycin). The distribution of green fluorescence dots was observed under fluorescence microscopy after incubation in the thermotank for 48 h. Cells were lysed by Trizol and RT-PCR was conducted to detect mRNA expression of autophagy marker protein LC3-II in MFC cells.

### 2.11. Western Blot to Analyze Effects of WK on the MAPK Signaling Pathway in MFC and BGC-823 Cells

With the same dose of distilled water as the control group, WK extracts of different concentrations (10 mg/mL, 20 mg/mL, and 40 mg/mL) were incubated with gastric cancer cells for 48 hours. The cell lysis occurred on ice and related protein was obtained. Western blot was used to determine the expression of ERK, JNK, and p38 proteins in the MAPK signaling pathway of MFC and BGC-823 cells.

### 2.12. Antitumor Effect of WK on Gastric Cancer Model Mice

A mouse gastric cancer cell line MFC was grafted into 40 ICR mice. The mice were randomly divided into 5 groups: the blank control group (control) where the model mice were injected with normal saline through the tail vein; the WK extracts (WK) group in which WK of different concentrations were given by gavage: 9.75 g/kg (low dose), 19.5 g/kg (medium-dose), and 39 g/kg (high-dose); and the positive control group (cisplatin) where the mice underwent an intraperitoneal injection of cisplatin 5 mg/kg.

The longest diameter (a) and shortest meridian (b) of axillary tumors were measured with digital calipers on days 1, 3, 5, 7, 9, and 11. The volume of transplanted tumor was calculated according to the formula: V (mm^3^) = *πab*^2^/6, and a graph of tumor growth was drawn.

The mice were euthanized after a 10-day administration. The transplanted tumors were stripped and weighed. The tumor inhibition rate (TIR) was calculated according to the following formula: TIR (%) = (C − T/C) × 100%, where C means the average tumor weight of mice in model group and T represents the mean tumor weight of mice in the administration group.

### 2.13. Western Blot to Analyze Effects of WK on the MAPK Signaling Pathway in Gastric Cancer Model Mice

The tumor tissue was quickly stripped from euthanized mice and was stored at −80°C for later use. The tumor tissue was lysed to obtain protein samples. Western blot was used to detect the expression of ERK, JNK, p-p38, and p38 proteins in the MAPK signaling pathway.

### 2.14. Statistical Analysis

All data are processed by SPSS 19.0. The measurement data were compared with single factor analysis of variance. The homogeneity of variance was tested by the LSD method, and the heterogeneity of variance was tested by the DunnettsT3 method. *P* < 0.05 was considered statistically significant.

## 3. Results

### 3.1. Active Ingredients of WK and Construction of “Active Ingredient-Target” Network

**Figure 1:**
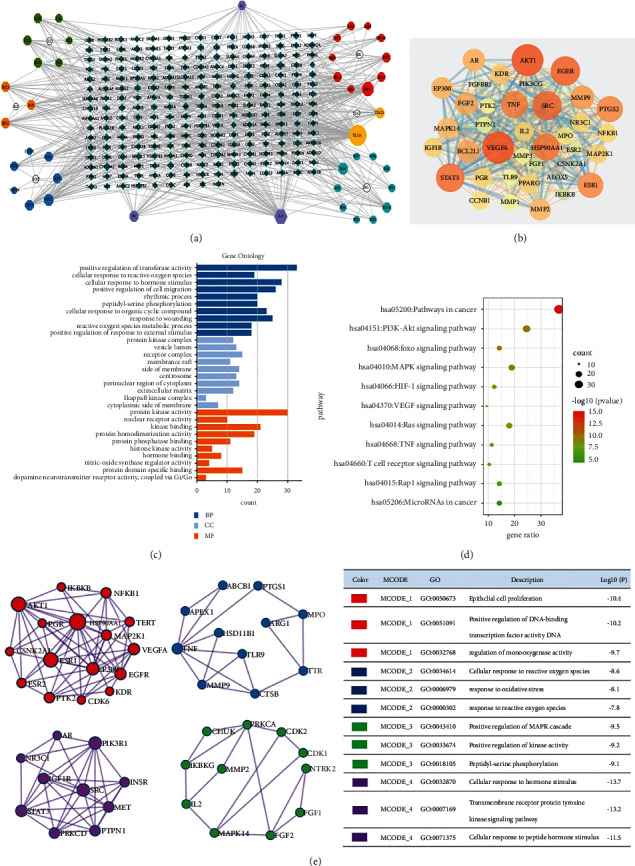
(a) “Active ingredient-target” network of WK. (b) PPI network of cross targets of WK and gastric cancer. (c) Bar graph of GO enrichment in the treatment of gastric cancer with WK. (d) Enrichment bubble chart of pathway for WK in gastric cancer treatment. (e) Internal potential module network and function description of the PPI network of WK in gastric cancer.

After preliminary screening, we found a total of 52 active ingredients in TCMSP. In addition, we supplemented 8 ingredients from the Chemistry Database [DB/OL] and checked relevant papers [12, 13] for 15 ingredients. After ADME screening, we finally got 41 active ingredients (See Table 1 in the Supplementary Material). Totally, there are 3 ingredients exist in more than 2 herbs, including stigmasterol, kaempferol, and beta-sitosterol. After prediction and screening by Swiss Target Prediction, 234 drug targets were finally obtained. The active ingredients and targets are imported into Cytoscape 3.7.2, and the “active ingredient-target” network is constructed. The network is composed of 281 nodes and 720 edges (Figure 1(a)).

### 3.2. Cross Targets of Gastric Cancer and WK

2978 and 298 targets for gastric cancer were retrieved from GeneCards and DisGeNET, respectively. After screening to remove duplicates, 1646 disease targets were finally obtained. Finally, we got 106 cross targets between WK and gastric cancer.

The cross targets were imported into string for prediction, medium confidence (0.0400) was chosen, the disconnected nodes were removed, and the results were imported into Cytoscape 3.7.2 for analysis. A PPI network was built finally (Figure 1(b)). The larger the node is, the darker the color is, and the higher the node degree is. The PPI network mainly includes VEGFA, AKT1, EGFR, STAT3, ERS1, etc. These targets may play an important role in the treatment of gastric cancer by WK.

### 3.3. Enrichment Analysis of Cross Targets

The cross targets were imported into Metascape to make GO and KEGG enrichment analyses. The top 10 results of BP, CC, and MF in GO were selected, ranking by Log10 (P) (Figure 1(c)). The results of pathway enrichment analysis were screened and tumor-related pathways were extracted (Figure 1(d)). The interaction relationship was analyzed with the molecular complex detection (MCODE) algorithm to make MCODE networks of BP. 4 netwoks were finally achieved; the biological process of WK in gastric cancer mainly appears to work to positively regulate DNA-binding transcription factor activity, which can influence the epithelial cell proliferation. In the process, MAPK cascade was also activited, and a series of kinases participated in this process (Figure 1(e)). The results showed that the main pathways for WK to treat gastric cancer may be the PI3K-Akt signaling pathway, MAPK signaling pathway, Ras signaling pathway, and VEGF signaling pathway etc. According to the information drawn from the result of MCODE networks and the conclusion of KEGG analysis, we think the MAPK signaling pathway is more likely to be the main pathway for WK to deal with gastric cancer. Stimulated by hormone, receptors on the cell surface transfer signaling into the cell from outside through the transmembrane receptor protein tyrosine kinase signaling pathway. Phosphorylate activates a series of protein kinases including mitogen-activated protein kinase (MAPK), which exhibits an antitumor effect by regulating the activity of protein kinases.

### 3.4. Molecular Docking Result of Active Ingredients to the Key Target Protein

**Figure 2:**
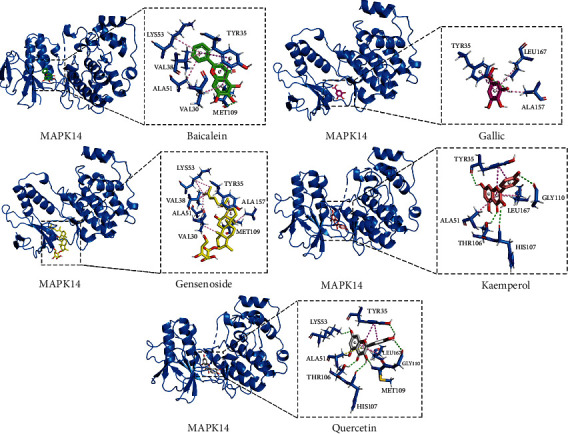
Binding mode of MAPK14 and top 5 active ingredients: Active site location: 22.528222 9.863593 32.015259 (defined by: centered on small molecule inhibitors in protein crystal complexes).

To explicit the relationship between the MAPK signaling pathway and WK, structures of MAPK14 (PDB : 2GFS) and the top 5 active ingredients (quercetin, kaempferol, baicalein, ginsenoside rh2, and gallic acid) were selected. The docking score ranked from −8.7 to −5.9, which shows a good binding activity (Table 2 in the Supplementary Material). The binding mode of MAPK14 and top 5 active ingredients is shown in Figure 2.

### 3.5. Effects of WK on the MFC Cell Viability

**Figure 3:**
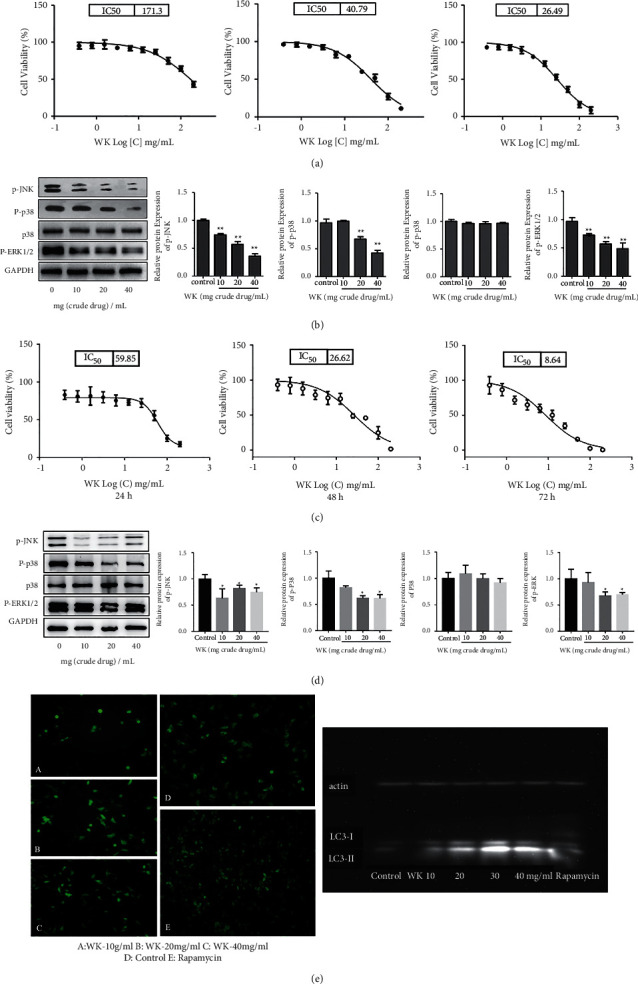
(a) Effect of WK on the viability of MFC cells. (b) Western blot and densitometric analysis of expression of P-JNK, P-p38, p38, P-ERK1/2, and GAPDH in MFC cells. ^*∗*^*P* < 0.05,^∗∗^*P* < 0.01 compared to the control group, which was considered statistically significant. (c) Effect of WK on the viability of BGC-823 cells. (d) Western blot and densitometric analysis of expression of P-JNK, P-p38, p38, P-ERK1/2, and GAPDH in BGC-823 cells. ^*∗*^*P* < 0.05 compared to the control group, which was considered statistically significant. (e) Effects of WK on the formation of autophagosome and the expression of biomarker protein LC3 in MFC cells.

After incubation with 0.39～200 mg/mL WK extracts for 24 h, 48 h, and 72 h, the cell viability of MFC cells was determined by CCK-8. As is shown in Figure 3(a), IC_50_ for 24 h is 171.3 mg/mL, IC_50_ for 48 h is 40.79 mg/mL, and IC_50_ for 72 h is 26.49 mg/mL. It can be seen that WK inhibits the proliferation of MFC cells in a dose-dependent and time-dependent manner.

### 3.6. Effects of WK on the MAPK Signaling Pathway in MFC Cells

After incubation with WK extracts (10 mg/mL, 20 mg/mL, and 40 mg/mL) for 48 h, Western blot was used to determine the expression of ERK, JNK, p-p38, and p38 proteins in the MAPK signaling pathway of MFC cells. It was found that compared with the blank control group, the expressions of p-JNK, p-p38, and p-ERR1/2 protein were significantly reduced, in a dose-dependent manner. While, WK had no significant effects on the expression of the p38 protein (Figure 3(b)).

### 3.7. Effects of WK on Cell Viability of the BGC-823

After BGC-823 cells were treated with 0.39～200 mg/mL WK extracts for 24 h, 48 h, and 72 h, the cell viability was determined by CCK-8. The results of the experiment are shown in Figure 3(c). IC_50_ for 24 h is 59.85 mg/mL, IC_50_ for 48 h is 26.62 mg/mL, and IC_50_ for 72 h is 8.64 mg/mL. It can be seen that WK inhibits the proliferation of BGC-823 cells in a dose-dependent and time-dependent manner.

### 3.8. Effects of WK on the MAPK Signaling Pathway in BGC-823 Cells

After the WK extracts (10 mg/mL, 20 mg/mL, and 40 mg/mL) were incubated with BGC-823 cells for 48 h, we used Western blot to determine the expression of p-JNK, p-p38, p38, p-ERK1/2, and GAPDH in BGC-823 cells. It was found that compared with the blank control group, the expressions of p-JNK, p-p38, and p-ERR1/2 proteins in the MAPK signaling pathway were significantly reduced, in a dose-dependent manner. While, WK had no significant effects on the expression of the p38 protein (Figure 3(d)).

### 3.9. Effects of WK on Autophagy of MFC Cells

Under the fluorescence microscope, the autophagosome green punctate fluorescent particles were evenly scattered in the cytoplasm of the control group. While, the positive staining around the nucleus and the aggregation of fluorescent particles could be observed in the cytoplasm of WK groups, which is similar to the positive control group (Rapamycin). According to PCR, the expression of LC3-II mRNA increased significantly in WK groups compared to the control group, which is positively correlated to the concentration of WK. The abovementioned results suggest that WK can induce autophagy of MFC cells in a dose-dependent manner (Figure 3(e)).

### 3.10. Antitumor Efficacy of WK in MFC-Transplanted Tumor Mice

**Figure 4:**
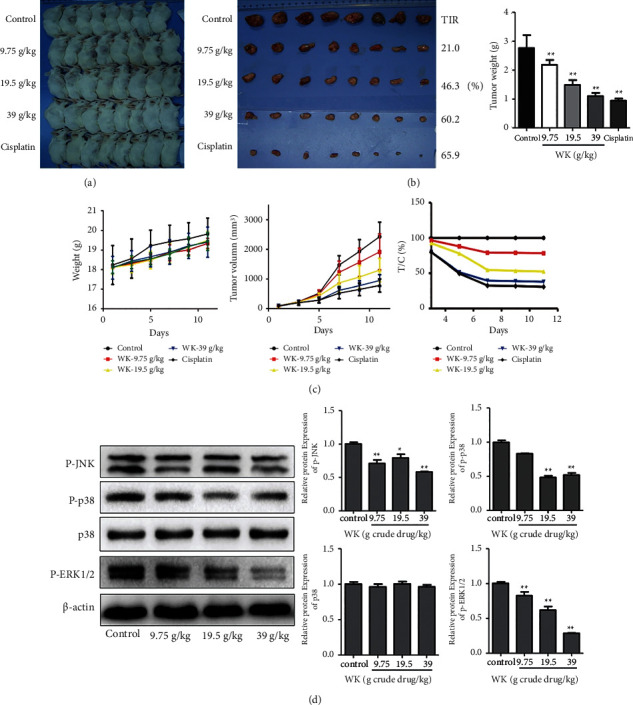
Antitumor efficacy and the effect of WK on the MAPK signaling pathway in mice with a transplanted tumor. (a) (b) Images of mice in each group and transplanted tumors, TIR, and tumor weight of each group. ^∗∗^*P* < 0.01 compared to control group, which was considered statistically significant. (c) Weight and volume of the transplanted tumor in ICR mice. (d) Western blot and densitometric analysis of the expression of P-JNK, P-p38, p38, P-ERK1/2, and *β*-action in ICR mice. ^*∗*^*P* < 0.05,^∗∗^*P* < 0.01 compared to control group, which was considered statistically significant.

As is shown in Figure 4(c), the growth of tumor volume accelerated in each group from day 5, especially in the control group. While, the growth rate of WK-39 g/kg and cisplatin group was relatively slow, and the tumor volume of the cisplatin group was smallest. TIR of the WK-low dose, WK-medium dose, WK-high dose, and cisplatin group are 21.0%, 46.3%, 60.2%, and 65.9%, respectively (Figure 4(b)). TIR of WK groups shows a dose-effect relationship, and the effect of high-dose group is nearly close to the cisplatin group.

### 3.11. Effects of WK on the MAPK Signaling Pathway in ICR Mice

As is shown in Figure 4(d), compared with control Group, WK reduced the expression of p-ERK1/2 in tumor tissues of the ICR mice, which is dose-dependent. The effects of p-JNK and p-p38 are not completely dose-dependent. In addition, WK has no significant effects on the expression of p38.

## 4. Discussion

The incidence of gastric cancer has been rising in recent years. However, conventional chemotherapy and radiotherapy limited in advanced gastric cancer. Although new drugs such as targeted therapy and immune checkpoint inhibitors show certain efficacy in advanced gastric cancer, the five-year survival rate remains low [14–16]. Besides, the adverse reactions such as digestive tract reaction and bone marrow inhibition are still challenges in gastric cancer treatment. Moreover, advanced gastric cancer patients suffer from weight loss and dyscrasia, which makes it hard for them to tolerate antitumor treatment. With low toxic side effects, traditional Chinese medicine (TCM) or Chinese patent medicines provide an effective treatment for advanced gastric cancer patients. Even though our previous research proved that WK could exert antitumor effects by inducing autophagy to cause death of gastric cancer cells, the specific pathways and mechanisms remain to be proved [2].

As an emerging discipline, network pharmacology integrates system biology, bioinformatics, and high-throughput histology. On the basis of drug and disease targets, network pharmacology predicts multiple mechanisms of drugs through a series of algorithms, providing a preliminary basis for drug research. TCM and its compound prescriptions have complex components and targets, and they often participate in the treatment of diseases through various pathways [17]. According to PPI network results, key targets of WK in the treatment of gastric cancer are likely to be VEGFA, AKT1, EGFR, STAT3, ERS1, HSP90AA1, etc. Based on the enrichment analysis of GO and KEGG pathways, we believe that the PI3K-Akt signaling pathway, MAPK signaling pathway, Ras signaling pathway, and VEGF signaling pathway are the main pathways participating in the gastric cancer treatment of WK. According to GO-BP analysis in the module, the 4 MCODE networks of BP shows a series of kinases in MAPK cascade participating in the biological process, which may have a connection with positive regulation of DNA-binding transcription factor activity and epithelial cell proliferation. All things considered, although the PI3K-Akt signaling pathway shows a higher log10 (*P* value), we tend to choose the MAPK signaling pathway as the key pathway in cancer treatment of WK [18]. To confirm our speculation, we also conducted a molecular docking to verify the interaction between active ingredient and key target protein MAPK14 in the MAPK signaling pathway, which shows a good binding activity.

As an important bridge from extracellular signals to intracellular responses, the MAPK pathway is considered as a key pathway related to tumor cell growth. Epidermal growth factor (EGF) and epidermal growth factor receptor (EGFR) participate in the construction of the MAPK signaling pathway. When stimulated by hormones, the growth factors (GFs) of the MAPK signaling pathway bind to transmembrane glycoproteins of the receptor tyrosine kinase (RTK) family and activate its signal transduction cascade. It promotes the development of RAS/RAF/MEK and ERK1/2 pathway. The dual phosphorylation activation of MAPK can phosphorylate some transcription factors, initiate the transcription and translation of response genes, regulate protein kinase activity, and participate in various physiological processes such as cell growth, differentiation, apoptosis, and death [18]. Furthermore, VEGF has been confirmed to take part in the regulation of PI3K/Akt and MAPK signaling pathways, which are related to tumor angiogenesis [19]. Among them, VEGFA is related to extracellular signal-regulated kinases (ERKs), phosphoinositide 3 kinase (PI3K)/Akt and p38 mitogen-activated protein kinase (MAPK), which participates in angiogenesis and vascular permeability regulation by regulating these kinases [20]. VEGFA-targeted drugs, such as bevacizumab, ranibizumab, etc., also make some achievements in clinic. STAT3 is related to inflammatory effect and apoptosis process, and it is believed to play a key role in cancer progression, angiogenesis, metastasis, and drug resistance. EGF can induce STAT3 activation and block the interaction between STAT3 and EGFR, which can inhibit tumor growth [21, 22].

Although several key targets predicted by network pharmacology tend to work in both PI3K-Akt and MAPK signaling pathways, the module analysis based on MCODE about GO-BP indicated that the biological process involved more kinases connected with the MAPK pathway. The result shows more preference for the MAPK signaling pathway. Besides, many studies have proved that the herbs consisting WK could activate the PI3K-Akt signaling pathway [23, 24]. As a Chinese herbal compound prescription, WK takes effects based on the truth that each herb works respectively. We can even get the speculation that WK can work in the PI3K-Akt pathway without further proof. For the reasons mentioned above, we choose to focus on the MAPK signaling pathway to explore the antitumor mechanism of WK in this study. As the relevant targets predicted by network pharmacology is not the specific target of MAPK signaling pathway, we selected the classic protein subgroups ERK, JNK, and p38 of the MAPK signaling pathway for verification in vitro and vivo experiments. According to cell and animal experiment results, WK could lower p-JNK, p-p38, and p-ERR1/2e expressions, which indicated that the MAPK signaling pathway participated in antitumor effects of WK. It is confirmed that WK has a clear antitumor effect on gastric cancer via the MAPK signaling pathway. This provides a further theoretical basis for the application of WK in the clinical treatment of gastric cancer.

However, in this study, the data in vitro experiment show that the antitumor effect of the WK high-dose group is close to that of platinum. According to clinical experience, it is almost impossible that TCM has an equivalent effect with platinum-based chemotherapy. We use a mouse gastric cancer cell line MFC to make tumor transplantation in ICR mice, which may differ from a human gastric cancer cell line. Moreover, ICR mice have a high heterozygous rate, which may cause individual differences. Therefore, the result of animal experiment cannot be extrapolated to human beings directly. Moreover, the concentration of the high-dose group is higher than routine dosage in clinic; this may also cause the strong antitumor effect. WK is a prescription summarized from clinical experience, which is usually used in combination with chemotherapy to enhance the antitumor effect of chemotherapy, and its antineoplastic effects have also been observed in clinic. In vivo and vitro experiments, we also proved that WK has a good inhibitory effect on gastric cancer cells. The tumor inhibition rate (TIR) of clinical routine dosage (medium-dose group) in tumor bearing mice (close to 50%), even is lower than the high-dose group, is also obvious enough to prove the antitumor potential of WK.

This study also has some limitations. We only explored one pathway that we think is more likely to work. As we did not verify other possible pathways, we cannot verify whether WK can participate in other pathways to make a synergistic antitumor effect. In addition, we only proved that autophagy existed when gastric cancer cells are treated with WK. Whether there is a connection with the MAPK signaling pathway is still underway to be verified in further experiments.

In this study, we explored the potential mechanism of WK in gastric cancer with network pharmacology and predicted possible pathways with a higher probability. Then, we verified that WK displayed an antitumor effect in gastric cancer by participating in the MAPK signaling pathway, and the antitumor effect has a dose dependency, laying a certain theoretical foundation for further exploration to expand the application of WK.

## Data Availability

The data used to support the findings of this study are available from the corresponding author upon request.

## Supplementary Materials

Supplementary Materials Supplementary Materials. Supplementary 1. Table 1 Effective Ingredients of WK. Supplementary 2. Table 2 Docking score of MAPK14 and top 5 active ingredients.
