# Supplementary material for: Exploring the Mechanism of Weikang Keli in Inhibiting Gastric Cancer through the MAPK Signaling Pathway: Based on Network Pharmacology and Experimental Verification
Source: Evid Based Complement Alternat Med. 2022 May 2;2022:2662288. doi: 10.1155/2022/2662288 (PMC9085321; doi:10.1155/2022/2662288)
Supplement: Supplementary Materials — Supplementary 1. Table 1 Effective Ingredients of WK. Supplementary 2. Table 2 Docking score of MAPK14 and top 5 active ingredients. [file 2662288.f1.zip › 2662288.f1/Supplementary material -Table 2 [1].docx]

| Protein | Complound | Score | | H-bond | Hydrophobic interactions |
| --- | --- | --- | --- | --- | --- |
| MAPK14 | baicalein | -8.7 | MET109 | | TYR35, VAL30, VAL38, LYS53, ALA51 |
|  | gallic acid | -5.9 | TYR35 | | ALA57, LEU167 |
|  | ginsenoside rh2 | -6.2 |  | | VAL30, VAL38, ALA51, LYS53, TYR35, MET109, ALA157 |
|  | kaempferol | -7.9 | TYR35, GLY110, HIS107, THR106 | | TYR35, ALA51, LEU167 |
|  | quercetin | -8.2 | TYR35, GLY110, MET109, HIS107, THR106, LYS53, | | TYR35, ALA51, LEU167 |

Table 2 Docking score of MAPK14 and top 5 active ingredients
