# Supplementary material for: Exploring the Mechanism of Weikang Keli in Inhibiting Gastric Cancer through the MAPK Signaling Pathway: Based on Network Pharmacology and Experimental Verification
Source: Evid Based Complement Alternat Med. 2022 May 2;2022:2662288. doi: 10.1155/2022/2662288 (PMC9085321; doi:10.1155/2022/2662288)
Supplement: Supplementary Materials — Supplementary 1. Table 1 Effective Ingredients of WK. Supplementary 2. Table 2 Docking score of MAPK14 and top 5 active ingredients. [file 2662288.f1.zip › 2662288.f1/Supplementary Materials-Table 1 Effective Ingredients of WK.doc]

Table 1 Effective Ingredients of WK

| Nunber | MolName | Nunber | MolName | Nunber | MolName |
| --- | --- | --- | --- | --- | --- |
| RS1 | Diop | EZ5 | monodemethylcurcumin | BZ3 | 8β-ethoxy atractylenolide Ⅲ |
| RS2 | Celabenzine | EZ6 | Keto-curcumin | TLG1 | quercetin |
| RS3 | Dianthramine | EZ7 | allyl curcumin | TLG2 | sitosterol |
| RS4 | arachidonate | BX1 | 24-Ethylcholest-4-en-3-one | HJT1 | rhodioloside |
| RS5 | Frutinone A | BX2 | Cavidine | HJT2 | gallic acid |
| RS6 | ginsenoside rh2 | BX3 | baicalein | HJT3 | caffeic acid |
| RS7 | Girinimbin | BX4 | Baicalin | HJT4 | tyrosol |
| RS8 | Panaxadiol | BX5 | coniferin | HJT5 | 4-Hydroxybenzoic acid |
| RS9 | alexandrin_qt | BX6 | 10,13-eicosadienoic | HJT6 | p-Cresol |
| RS10 | Fumarine | BX7 | (3S,6S)-3-(benzyl)-6-(4-hydroxybenzyl)piperazine-2,5-quinone | HJT7 | 3,4-Dihydroxy-5-methoxybenzoic acid |
| EZ1 | hederagenin | BX8 | Cycloartenol | A1 | Stigmasterol |
| EZ2 | bisdemethoxycurcumin | BX9 | beta-D-Ribofuranoside, xanthine-9 | A2 | kaempferol |
| EZ3 | demethoxycurcumin | BZ1 | α-Amyrin | B1 | beta-sitosterol |
| EZ4 | bisdemethoxycurcumin | BZ2 | (3S,8S,9S,10R,13R,14S,17R)-10,13-dimethyl-17-[(2R,5S)-5-propan-2-yloctan-2-yl]-2,3,4,7,8,9,11,12,14,15,16,17-dodecahydro-1H-cyclopenta[a]phenanthren-3-ol | | |
